# Dysphagia in the Context of a Serious Systemic Disease

**DOI:** 10.1007/s00455-020-10155-6

**Published:** 2020-07-22

**Authors:** Florentina Sophie Ferstl, Simon Peter Gampenrieder, Felix Renneberg, Sebastian Roesch

**Affiliations:** 1 Department of Otorhinolaryngology, Head and Neck Surgery, Paracelsus Medical University Salzburg, Austria, Muellner Hauptstrasse 48, 5020 Salzburg, Austria; 2 Department of Internal Medicine III with Haematology, Medical Oncology, Haemostaseology, Infectiology and Rheumatology, Oncologic Center, Salzburg Cancer Research Institute - Laboratory for Immunological and Molecular Cancer Research (SCRI-LIMCR), Paracelsus Medical University Salzburg, Muellner Hauptstrasse 48, 5020 Salzburg, Austria

## Case Description

**Fig. 1:**
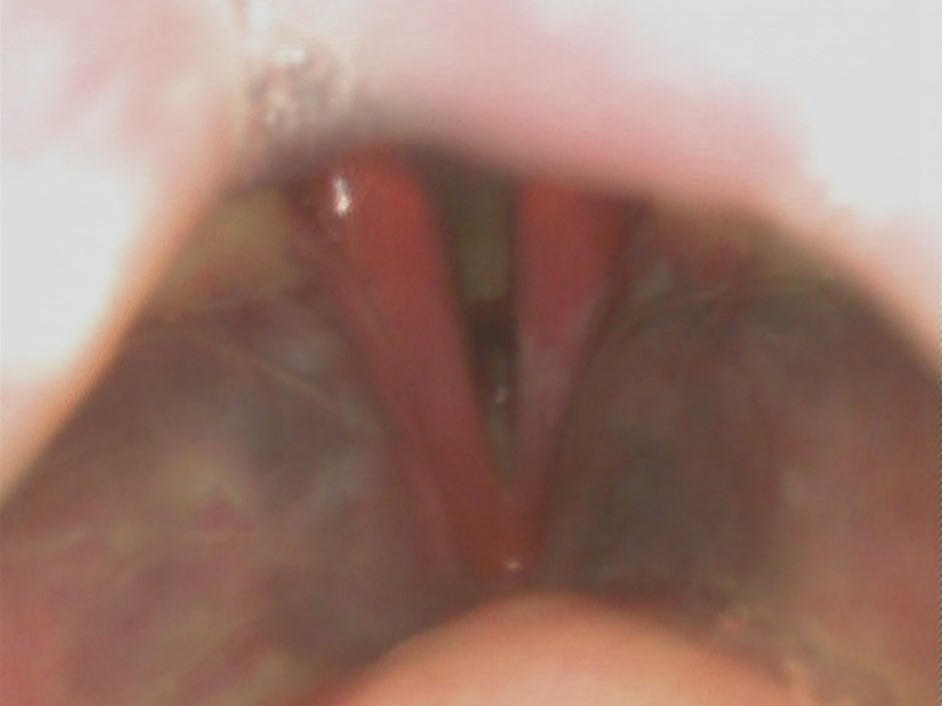
Endoscopic picture of laryngeal space during first examination on admission

**Fig. 2:**
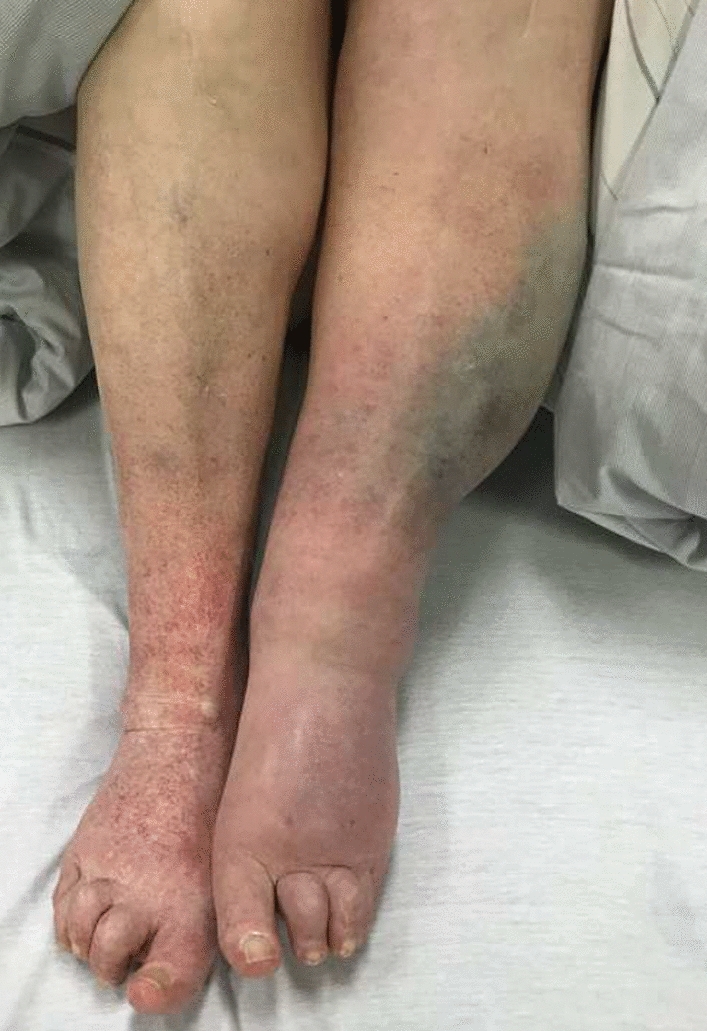
Local hematoma and swelling of the left lower leg and petechia of the right ankle around six hours after admission

A 78-year-old, female patient was referred to the department of otolaryngology due to subjective difficulties during swallowing and neck stiffness for two weeks. Primary clinical investigation revealed a superficial hematoma below the right mandible, not significant in size and without any history of recent trauma. During transnasal endoscopic examination of the nose, pharynx and larynx, a hematoma of the mucosa was detected. The hematoma was sharply limited to the vestibular folds and the vocal folds on both sides and did not lead to relevant swelling or narrowness (Fig. 1). There was no accumulation of saliva, neither any sign of a foreign body. Agility of both vocal folds was not reduced to any extend. Hoarseness was clinically not significant, breathing was normal in frequency and there was no stridor, not even during forced breathing. Besides a changed color of the mucosa due to the hematoma, there was no sign of injury or suspicious mass. The patient was admitted to the in-patient clinics for further observation. Initial lab testing revealed leukocytosis (12,090/µl; upper limit of normal [ULN] 9000/µl), thrombocytosis (429,000/µl; ULN 400,000/µl) and mildly reduced hemoglobin (10.2 g/dl; lower limit of normal [LLN] 12.0 g/dl). C-reactive protein as well as neutrophils were elevated with 16.4 mg/dl (ULN 0.5 mg/dl) and 77.8% (ULN 70%), respectively. A subsequent contrast-enhanced computed tomography of the neck excluded acute arterial bleeding or a submucosal mass. Within 6 h after admission, the patient complained about a sudden and severe pain of the left leg. Clinical examination revealed considerable swelling due to a hematoma in the left lower leg as well as petechia of the right ankle without swelling (Fig. 2). Immediate angiological consultation excluded thrombosis of the deep veins by duplex sonography, however, the swelling increased constantly and visibly. The patient was transferred to the department of orthopedics and traumatology, where surgical therapy was indicated because of imminent compartment syndrome. Fiber optic intubation was performed under attendance of an otolaryngologist, without further complications and without signs of further laryngeal swelling.

## Answer

The clinical appearance of spontaneous, rapidly developing hematomas raised the suspicion of a coagulation disorder, despite the negative family history in this regard. Significant prolongation of the activated partial thromboplastin time (aPTT = 64 s, ULN 38 s) with normal prothrombin time (PT) and negative medical history for anticoagulant agents led to the tentative diagnosis of acquired hemophilia. A completely suppressed factor VIII activity (< 1%; LLN 50%) and the detection of high factor VIII inhibitor titers (31 Bethesda Units [BU]/ml; ULN < 1 BU/ml), in synopsis with the characteristic symptom of a spontaneous, unreproducible and first-time bleeding event in this patient, confirmed the diagnosis of acquired hemophilia A (AHA), finally. Hemostatic therapy with activated prothrombin complex concentrates (APCC) was initiated right after surgery and immunosuppression with prednisolone was started subsequently. The patient remained intubated until bleeding was under control. Postoperative lab control revealed a hemoglobin decline to 7.4 g/dl. Unfortunately, several wound debridements at the leg were indicated in this patient, requiring prolonged hemostatic therapy with APCC, followed by recombinant factor VIIa (rFVIIa) and recombinant porcine FVIII (rpFVIII). Under this treatment, no new mucosal bleedings occurred and the initial hematoma in the larynx did not increase, so that the patient could by extubated on the second day after surgery. After 1 month of intensive immunosuppressive therapy with prednisolone, rituximab and one immunoadsorption, the factor VIII increased to normal values and no further substitution was needed until the patient was discharged.

## Discussion

AHA is a rare autoimmune bleeding disorder characterized by autoantibodies directed against coagulation factor VIII [1]. It is associated with autoimmune diseases, infections and malignancies, however, about 50% remain idiopathic [2]. The two pillars of therapy are (a) anti-hemorrhagic treatment with bypassing agents (APCC or rFVIIa) or recombinant porcine factor VIII concentrate and (b) inhibitor eradication with immunosuppressive therapy (mostly corticosteroids, cyclophosphamide and rituximab) [1]. The clinical symptom of dysphagia may result from a great variety of causes, mainly neuromotor abnormalities, in the course of neurological disease, after trauma, after chemoradiation or surgery and structural changes caused by tumors or cervical spine disorders [3]. In case of causal coagulation disorders, clinically obvious obstruction of the airway sensitizes the examiner in most cases [4]. However, even initially harmless appearing findings like in this patient, may lead to critical illness, claiming for careful diagnostics and rapid interdisciplinary treatment. AHA is easy to diagnose (bleeding event plus prolonged aPTT with normal PT [1]) and should be considered, despite its rareness, as one possible cause of atypical hematoma or postoperative bleeding.
